# White blood cell count profiles in anti-aquaporin-4 antibody seropositive neuromyelitis optica spectrum disorder and anti-myelin oligodendrocyte glycoprotein antibody-associated disease

**DOI:** 10.1038/s41598-023-33827-3

**Published:** 2023-04-20

**Authors:** Tetsuya Akaishi, Tatsuro Misu, Kazuo Fujihara, Kumi Nakaya, Naoki Nakaya, Tomohiro Nakamura, Mana Kogure, Rieko Hatanaka, Fumi Itabashi, Ikumi Kanno, Kimihiko Kaneko, Toshiyuki Takahashi, Juichi Fujimori, Yoshiki Takai, Shuhei Nishiyama, Tadashi Ishii, Masashi Aoki, Ichiro Nakashima, Atsushi Hozawa

**Affiliations:** 1 Department of Neurology, Tohoku University Graduate School of Medicine, Seiryo-Machi 1-1, Aoba-Ku, Sendai, Miyagi 980-8574 Japan; 2 Department of Education and Support for Regional Medicine, Tohoku University Hospital, Sendai, Japan; 3 Department of Multiple Sclerosis Therapeutics, Fukushima Medical University, Fukushima, Japan; 4 Tohoku Medical Megabank Organization, Tohoku University, Sendai, Japan; 5 Department of Neurology, National Hospital Organization Yonezawa National Hospital, Yonezawa, Japan; 6 Department of Neurology, Tohoku Medical and Pharmaceutical University, Sendai, Japan

**Keywords:** Neurology, Neurological disorders

## Abstract

White blood cell (WBC) count profiles in anti-aquaporin-4 antibody-positive neuromyelitis optica spectrum disorder (AQP4-NMOSD) and anti-myelin oligodendrocyte glycoprotein antibody-associated disease (MOGAD) are still unknown. This study evaluated the total WBC count, differential WBC counts, monocyte-to-lymphocyte ratio (MLR), and neutrophil-to-lymphocyte ratio (NLR) in patients with these diseases within three months from an attack before acute treatment or relapse prevention and compared the profiles with those in matched volunteers or in multiple sclerosis (MS) patients. AQP4-NMOSD patients (n = 13) had a higher neutrophil count (p = 0.0247), monocyte count (p = 0.0359), MLR (p = 0.0004), and NLR (p = 0.0037) and lower eosinophil (p = 0.0111) and basophil (p = 0.0283) counts than those of AQP4-NMOSD-matched volunteers (n = 65). Moreover, patients with MOGAD (n = 26) had a higher overall WBC count (p = 0.0001), neutrophil count (p < 0.0001), monocyte count (p = 0.0191), MLR (p = 0.0320), and NLR (p = 0.0002) than those of MOGAD-matched volunteers (n = 130). The three demyelinating diseases showed similar levels of the total and differential WBC counts; however, MOGAD and MS showed different structures in the hierarchical clustering and distributions on a two-dimensional canonical plot using differential WBC counts from the other three groups. WBC count profiles were similar in patients with MOGAD and MS but differed from profiles in matched volunteers or patients with AQP4-NMOSD.

## Introduction

Anti-aquaporin-4 antibody (AQP4-IgG)-positive neuromyelitis optica spectrum disorder (NMOSD), anti-myelin oligodendrocyte glycoprotein antibody-associated disease (MOGAD), and multiple sclerosis (MS) are distinct autoimmune-related relapsing neurological diseases of the central nervous system (CNS)^1–3^. Lesions associated with these diseases may involve any part of the brain, optic nerve, or spinal cord^4,5^. Efficient relapse prevention, including immunosuppressants and monoclonal antibodies administration, is essential as the neurological disability in patients with these diseases often progresses due to relapses^6–9^. Many of the currently available effective relapse prevention strategies affect circulating lymphocytes, complement activation pathways, cytokines, and chemokines. This implies the impact of peripherally circulating white blood cells on the development of these diseases^10–13^. It has been recently reported that white blood cell (WBC) count profiles in patients with MS are different from those in matched healthy volunteers and are characterized with elevated neutrophil, monocyte, and basophil counts^14^. However, the exact profiles of the total and differential white blood cell (WBC) counts in AQP4-IgG-positive NMOSD and MOGAD, for which disease concept has been recently established, is not determined yet. Elucidating detailed WBC count profiles in these diseases will help us to understand their mechanisms and find appropriate therapeutic strategies. Therefore, in this study, we evaluated the total and differential WBC counts in patients with these diseases and compared them with counts in matched volunteers or patients with MS to understand the abnormalities and characteristics of WBC count profiles in AQP4-IgG-positive NMOSD and MOGAD.

## Methods

### Study design

This study aimed to clarify the WBC count profiles of patients with AQP4-IgG-positive NMOSD and MOGAD during the acute to subacute phases before treatment. WBC count profiles were collected from patients with AQP4-IgG-positive NMOSD or MOGAD within three months from the last clinical attack before starting acute treatment (e.g., steroid pulse therapy, plasma exchange) or relapse prevention. The control group included age- and sex-matched volunteers for each disease. Matching was performed using propensity scores calculated according to age and sex. Moreover, data from patients with MS before starting acute treatment or disease-modifying drugs were also collected to further clarify the characteristics of WBC count profiles in patients with AQP4-IgG-positive NMOSD and MOGAD. All blood test data were collected during the patients’ first visit to Tohoku University Hospital, Japan, between April 2013 and March 2022. Patients whose first blood tests were performed before April 2013 were not enrolled as the current automated hematology analyzer was introduced at the university hospital in April.

### Participants

Eligible patients with each of the afore-mentioned diseases were enrolled before the initiation of any acute or chronic treatment for neurological diseases. Patients who had already initiated immune-modulatory agents or disease-modifying drugs at the time of blood tests were excluded. All enrolled patients with MS met the latest version of the McDonald diagnostic criteria^15^, and all enrolled patients with AQP4-IgG-positive NMOSD and MOGAD met the international consensus on the diagnosis of these diseases^1^. From the initially recruited patients, a woman in her 30 s with MS (WBC: 13,600 /μL) was excluded because of a recent history of trauma with active inflammation at the time of blood test. Age- and sex-matched volunteers with 1:5 matching (i.e., one patient to five volunteers) were prepared for each of the two neurological diseases using propensity score matching. Age- and sex-matched volunteers were enrolled from a database of volunteers registered in the Tohoku Medical Megabank Organization (ToMMo) Community-Based Cohort Study since 2013. The detailed profiles of the volunteers have been previously reported^16,17^.

### Evaluated variables

Demographic data including age and sex were obtained. Furthermore, the total WBC count [/μL], and the differential counts of neutrophils, eosinophils, basophils, lymphocytes, and monocytes were obtained from the blood test data. According to these differential counts, the platelet-to-lymphocyte ratio (PLR), monocyte-to-lymphocyte ratio (MLR), and neutrophil-to-lymphocyte ratio (NLR) were further calculated. All patients with the three demyelinating diseases were assessed for the presence of serum AQP4-IgG and MOG-IgG. Microscopic live cell-based assays using AQP4-expressing or MOG-expressing HEK293 cells and Alexa Fluor 488-conjugated secondary antibodies were conducted to check for these antibodies in the serum^18,19^. Titers were calculated semi-quantitatively by performing serial two-fold dilutions.

### Statistical analysis

WBC count profiles were compared between patients with AQP4-IgG-positive NMOSD or MOGAD and the matched control group using the Mann–Whitney U (MWU) test. In addition, comparisons between the three disease groups (AQP4-IgG-positive NMOSD, MOGAD, and MS) were performed using the analysis of covariance (ANCOVA) with age and sex as covariates. Statistically significant differences between each pair of disease groups were evaluated using Tukey’s post-hoc tests. Furthermore, hierarchical clustering analyses were performed by using the five differential WBC counts for each group to evaluate their relationship. Violin and box-whisker plots were plotted for each group to visually evaluate and compare the distribution of WBC counts. Moreover, canonical plots based on the canonical structures of the five differential WBC counts were constructed to visually understand the different profiles of differential WBC counts between the diseases. The statistical significance was consistently set at p < 0.05 and was not adjusted for the number of multiple tests because of the exploratory nature of the present study^20^. Statistical and graph-building analyses were performed using JMP Pro version 14 (SAS, Cary, NC, USA).

### Ethics

This study was approved by the Institutional Review Board of the Tohoku University Graduate School of Medicine (approval number: 2020-4-072). It was performed in accordance with the current version of the Declaration of Helsinki, as revised in 2013, and the STROBE reporting guidelines for observational studies^21^. A written informed consent was obtained from all participants.

## Results

### Demographics of the participants

The number of enrolled participants for each disease group was as follows: 13 (2 male and 11 female) patients with AQP4-IgG-positive NMOSD, 26 (8 male and 18 female) patients with MOGAD, and 70 (17 male and 53 female) patients with MS. We initially enrolled 23,433 individuals with the full dataset of WBC count data from the ToMMo community-based cohort, in which the age- and sex-matched control groups were prepared. The number of the one-to-five-matched control group for each of AQP4-IgG-positive NMOSD and MOGAD was 65 (10 male and 55 female) volunteers in AQP4-matched control group and 130 (40 male and 90 female) volunteers in MOGAD-matched control group, respectively. The median and interquartile range (IQR; 25–75 percentiles) of the age at the time of the blood tests were 47 (39–57) years in the AQP4-IgG-positive NMOSD group, 43 (34.5–56) years in the MOGAD group, 33 (27–43) years in the MS group, 47 (39–57) years in the AQP4-matched control group, and 43 (34–57) years in the MOGAD-matched control group. All 13 patients with AQP4-IgG-positive NMOSD experienced their first clinical attack (i.e., onset episode) at the blood test, whereas 3 out of the 26 patients with MOGAD experienced an undiagnosed neurological episode (one with myelitis and two with optic neuritis) more than 10 years ago without subsequent relapse preventions.

### WBC count profiles between patients and matched HCs

**Figure 1:**
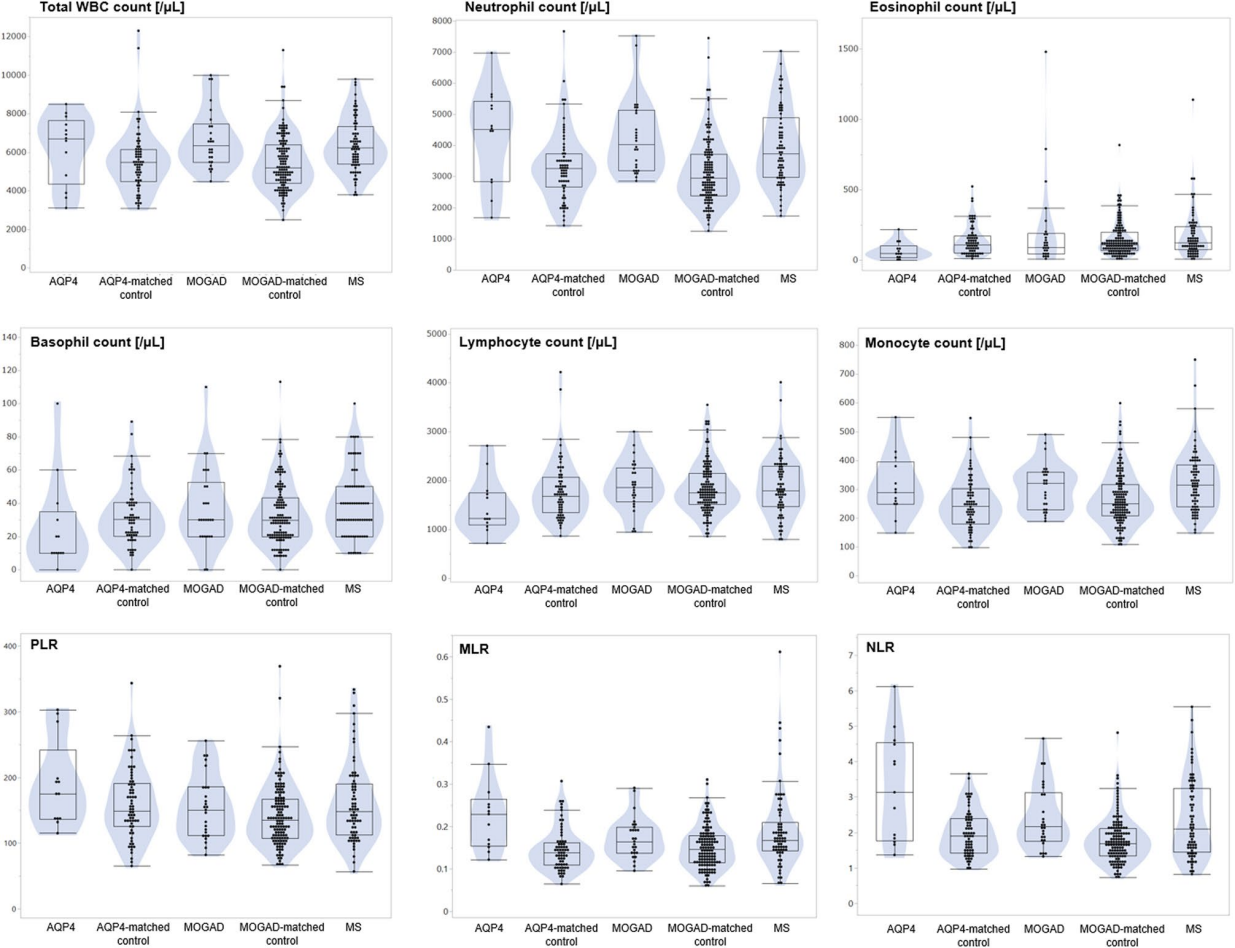
Distributions of the total and differential WBC counts in each group. Violin plots and box-whisker plots for the total and differential WBC counts, PLR, MLR, and NLR in each of the five groups are shown. The distributions of total WBC count, neutrophil count, monocyte count, MLR, and NLR were higher in patients with AQP4-IgG-positive NMOSD and MOGAD than in their matched volunteers as control groups. *AQP4* aquaporin-4, *MOGAD* anti-myelin oligodendrocyte glycoprotein antibody-associated disease, *NMOSD* neuromyelitis optica spectrum disorder, *MS* multiple sclerosis, *WBC* white blood cell.

The distributions of the total WBC counts, five differential WBC counts, PLR, MLR, and NLR in the evaluated disease groups are shown in Fig. 1. Statistically significant differences between the AQP4-IgG-positive NMOSD and the matched control groups were observed in the neutrophil count (p = 0.0247, MWU test), eosinophil count (p = 0.0111), basophil count (p = 0.0283), monocyte count (p = 0.0359), MLR (p = 0.0004), and NLR (p = 0.0037). Moreover, statistically significant differences between the MOGAD and the matched control groups were observed in the total WBC count (p = 0.0001), neutrophil count (p < 0.0001), monocyte count (p = 0.0191), MLR (p = 0.0320), and NLR (p = 0.0002). The results of the initial bivariate analyses for each evaluated WBC count between patients with AQP4-IgG-positive NMOSD or MOGAD and matched controls are shown in Table 1.

**Table 1:** White blood cell counts profiles between patients and matched healthy controls in AQP4-IgG-positive NMOSD and MOGAD.

	Patients	1:5 matched controls	P-values
AQP4-IgG-positive NMOSD (Patient group: n = 13 *vs* matched controls: n = 65)			
Total WBC count	6700 (4810–7440)	5500 (4500–6100)	0.0723
Hemoglobin [g/dL]	14.1 (13.6–14.5)	13.4 (12.7–14.2)	0.1955
Platelet count [10^3^/μL]	24.8 (21.4–31.2)	25.8 (22.3–29.0)	0.8986
Neutrophil [/μL]	4520 (2880–5280)	3260 (2710–3720)	0.0247
Eosinophil [/μL]	50 (20–80)	110 (50–170)	0.0111
Basophil [/μL]	10 (10–30)	30 (20–40)	0.0283
Lymphocyte [/μL]	1230 (1140–1720)	1690 (1350–2060)	0.0511
Monocyte [/μL]	290 (250–380)	240 (180–300)	0.0359
PLR	175.2 (138.5–198.3)	149.1 (125.8–190.0)	0.1315
MLR	0.229 (0.157–0.248)	0.139 (0.110–0.162)	0.0004
NLR	3.134 (1.835–4.485)	1.899 (1.438–2.381)	0.0037
MOGAD (Patient group: n = 26 vs matched controls: n = 130)			
Total WBC count	6350 (5550–7380)	5200 (4430–6350)	0.0001
Hemoglobin [g/dL]	13.8 (12.6–14.8)	13.7 (12.8–14.6)	0.8753
Platelet count [10^3/μL]	26.0 (22.9–31.6)	24.4 (21.0–28.1)	0.0578
Neutrophil [/μL]	4040 (3230–5090)	2960 (2390–3700)	< 0.0001
Eosinophil [/μL]	90 (60–190)	110 (70–200)	0.5927
Basophil [/μL]	30 (20–50)	30 (20–40)	0.3415
Lymphocyte [/μL]	1860 (1600–2220)	1750 (1520–2140)	0.4965
Monocyte [/μL]	320 (240–360)	250 (210–310)	0.0191
PLR	150.5 (113.1–185.0)	134.9 (107.7–166.5)	0.1799
MLR	0.164 (0.138–0.196)	0.146 (0.116–0.179)	0.0320
NLR	2.169 (1.788–3.072)	1.692 (1.355–2.108)	0.0002

Median and interquartile range (25–75 percentiles) for each variable are shown.

*AQP4* aquaporin-4, *MOGAD* anti-myelin oligodendrocyte glycoprotein antibody-associated disease, *NMOSD* neuromyelitis optica spectrum disorder.

### Results of ANCOVA adjusted for age and sex

Furthermore, the total WBC count, differential WBC counts, and derivatives were compared among the three disease groups (excluding the two matched control groups) after adjusting for age and sex using ANCOVA, followed by the Tukey’s post hoc test. As a result, there were no statistically significant differences in the total WBC count (p = 0.5864, ANCOVA), neutrophil count (p = 0.4236), eosinophil count (p = 0.2179), basophil count (p = 0.0995), lymphocyte count (p = 0.1225), monocyte count (p = 0.4597), PLR (p = 0.2685), MLR (p = 0.1713), and NLR (p = 0.0534). For reference, the Tukey’s post hoc test following ANCOVA between MS and AQP4-IgG-positive NMOSD groups in NLR showed statistical significance, with estimated least square means (95% CI) of 2.363 (2.084–2.642) for MS and 3.236 (2.593–3.879) for AQP4-IgG-positive NMOSD (p = 0.0415).

### Hierarchical cluster with the five differential WBC counts

**Figure 2:**
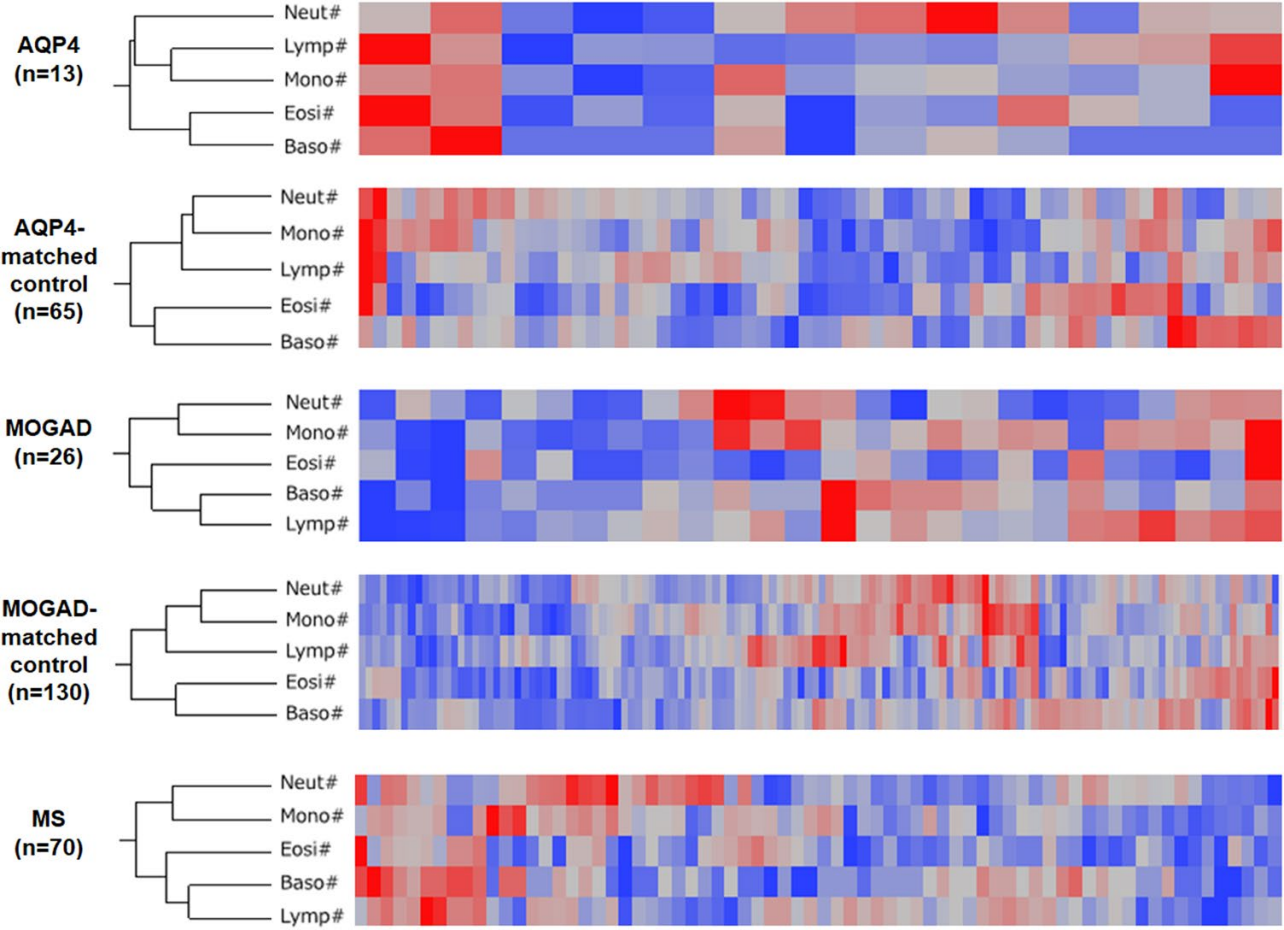
Hierarchical clustering for the five differential WBC counts in each group. Heat maps of the five groups were depicted using five differential WBC counts. Blood test data from each patient was aligned horizontally, and five differential WBC counts were aligned vertically. In the AQP4-IgG-positive NMOSD and the two control groups, eosinophil and basophil counts were aligned proximally within the same clade. In the MOGAD and MS groups, lymphocytes were aligned most proximal to the basophil count. *AQP4* aquaporin-4, *MOGAD* anti-myelin oligodendrocyte glycoprotein antibody-associated disease, *NMOSD* neuromyelitis optica spectrum disorder, *MS* multiple sclerosis, *WBC* white blood cell.

Moreover, hierarchical clustering analysis using the five differential WBC counts were performed for each group to visually evaluate their relationship. The cluster structures are shown in Fig. 2. They were roughly the same in the AQP4-IgG-positive NMOSD and the two control groups, with eosinophil and basophil counts aligned proximally within the same clade and away from the other three differential WBC counts. Meanwhile, the lymphocyte count was aligned most proximal to the basophil count in the MOGAD and MS groups, suggesting distinct profiles of differential WBC counts in them compared to those in the other three groups.

### Canonical plots based on differential WBC counts

**Figure 3:**
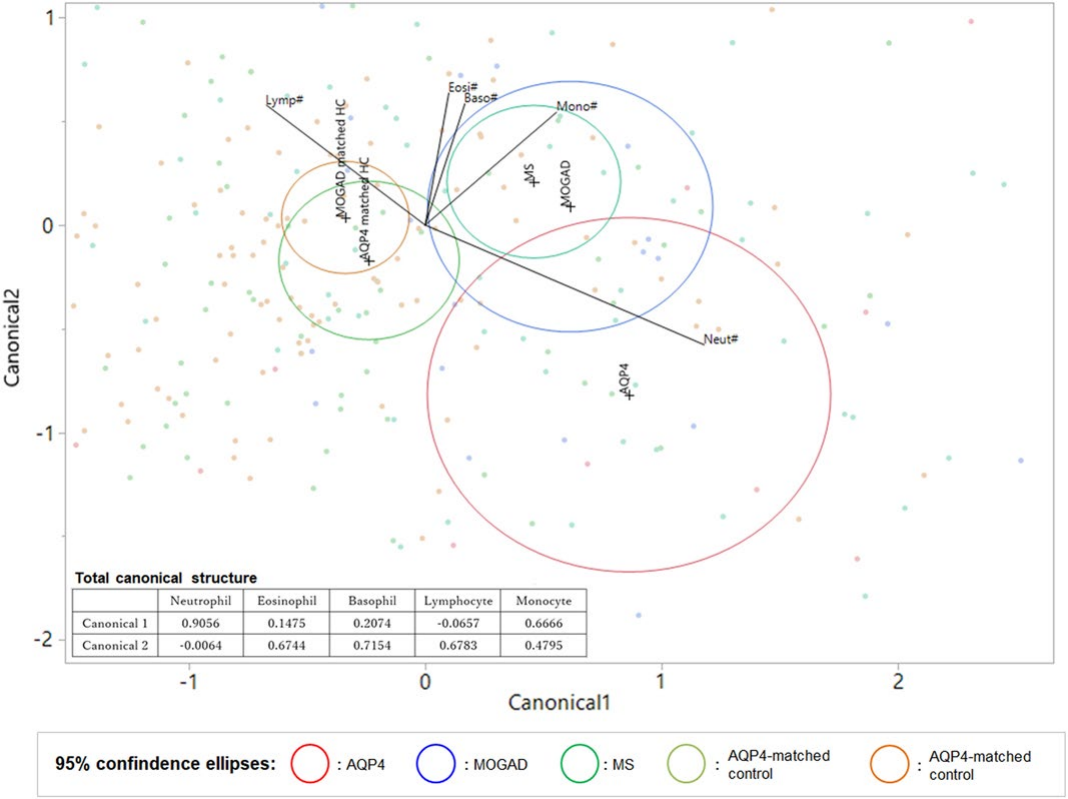
Two-dimensional canonical plots with the five differential white blood cell counts. In the two-dimensional canonical plot, 95% confidence ellipses for the means of the eight disease groups are shown. The ellipses for MOGAD and MS were located in approximately the same areas, whereas the ellipses for AQP4-IgG-positive NMOSD and the two matched controls were located away from them. *AQP4* aquaporin-4, *MOGAD* anti-myelin oligodendrocyte glycoprotein antibody-associated disease, *NMOSD* neuromyelitis optica spectrum disorder.

Canonical plots based on the five differential WBC counts were plotted for all five groups to further investigate the different WBC count profiles among them. The obtained two-dimensional canonical plot is shown in Fig. 3. The 95% confidence intervals for the means of the MOGAD and MS groups were located in approximately the same area. The ellipse for the AQP4-IgG-positive NMOSD group was located away from the ellipses for MOGAD, MS, and the two control groups. The distribution of plots in the AQP4-IgG-positive NMOSD group was mainly characterized by elevated neutrophil and decreased lymphocyte counts.

### Association between WBC count profiles and clinical profiles

Finally, correlations between WBC count profiles and clinical profiles in AQP4-IgG-positive NMOSD and MOGAD groups were comprehensively evaluated. These profiles included the serum titer levels of the disease-specific antibodies (AQP4-IgG and MOG-IgG), clinical phenotype of the neurological episode at the time of the blood test (optic neuritis, brain lesions, or myelitis), neurological disability level 1 year after the neurological episode in the blood test, and the presence of clinical relapses in the first 2 years after the neurological episode. None of the evaluated WBC count profiles, including MLR and NLR, were significantly correlated with the evaluated clinical profiles. Whether the correlations were evaluated using the Pearson’s R or the Spearman’s rho correlation coefficient, these results did not change. Furthermore, based on the marginally non-significant statistical results in the platelet count between the MOGAD and the matched control groups, the associations between the mean platelet volume (MPV) or platelet distribution width (PDW) and the evaluated clinical outcomes were additionally evaluated within each of the AQP4-IgG-positive NMOSD and MOGAD groups. As a result, neither MPV nor PDW showed significant correlations with any of the evaluated clinical outcomes in both of the two disease groups.

## Discussion

In this study, the total and differential WBC counts and their derivatives were compared between each of the groups with evaluated relapsing neurological diseases of the CNS (AQP4-IgG-positive NMOSD, MOGAD, and MS) and matched control groups. WBC count profiles were further investigated using hierarchical clustering analyses and two-dimensional canonical plots. The results suggested that the total WBC, neutrophil, and monocyte counts in patients with these diseases were higher than those in their matched controls. The structures of obtained tree with hierarchical clustering in each group implied that the lymphocyte count was more linked with the counts of eosinophils and basophils in the MOGAD and MS groups, whereas it was more linked with the counts of neutrophils and monocytes in the AQP4-IgG-positive NMOSD and two control groups. Moreover, the NLR and MLR values were higher in patients with AQP4-IgG-positive NMOSD and MOGAD than in their matched controls. Neutrophils and monocytes are innate immune cells with phagocytic and antigen-presenting roles. They both play pivotal roles in acute and chronic inflammatory responses against external pathogens and are associated with the mechanisms of some chronic diseases based on systemic inflammation, including MS^22–24^. In rodent models of MS, neutrophils have contributed to acute inflammation of the CNS through cytotoxicity and compromised blood–brain barrier, which result in the development of CNS lesions in animal models^25^. Circulating monocyte counts have predicted subsequent neurological disability levels in patients with MS^26^. Similar to neutrophils, monocytes secrete various cytokines and microbial factors^27^, thus attracting other immune cells to the focus of inflammation and contributing to chronic inflammation. Previous studies have implied that NLR and MLR may be better predictors for estimating disease activity or outcomes than simple counts of neutrophils and monocytes. NLR is associated with many autoimmune diseases, neurological diseases including MS, and malignancies^28–31^. This ratio has been reported to be linked to outcomes in some of these conditions, such as shorter survival in some malignancies and long-term neurological disability or relapse activity in MS^32–34^. MLR is also linked to the development of many autoimmune-related neurological diseases, including MS and Guillain-Barré syndrome^14,35^. Furthermore, MLR is associated with the outcomes of stroke and MS^32,36,37^. These findings collectively imply that both NLR and MLR are easily available markers for estimating inflammation levels in various chronic diseases, including many neurological diseases of the CNS. This study showed significantly different distributions in MLR and NLR between each of the disease groups and matched control group, but not in PLR. The platelet count, MPV, and PDW failed to show significant correlations with the evaluated clinical outcomes both in the AQP4-IgG-positive NMOSD and MOGAD groups. Although the circulating platelet count and the distribution of the size of platelets were not associated with the evaluated three neurological diseases in this study, a previous study reported that platelets are significantly activated in MS^38^. Additional assessment of platelet activities, such as plasma platelet microparticles and P-selectin expression, may benefit future researches evaluating potential roles of platelets in AQP4-IgG-positive NMOSD and MOGAD.

Although the total and differential WBC counts did not differ significantly among the three demyelinating disease groups, hierarchical clusters and canonical plots suggested different WBC count profiles between AQP4-IgG-positive NMOSD group and the other two disease groups. This may be incompatible with the current understanding of these three diseases. Generally, the clinical and radiological findings in MOGAD are thought to be more similar to the findings in AQP4-IgG-positive NMOSD than in MS. Furthermore, the cerebrospinal fluid cytokine profile in patients with MOGAD is more similar to the profile in those with AQP4-IgG-positive NMOSD than to the profile in those with MS, with a predominant representation of Th17-related cytokines such as IL-6, IL-8, and IL-17^39,40^. The different backgrounds of patients with different comorbid conditions and preceding clinical episodes heralding attacks in AQP4-IgG-positive NMOSD may be a possible explanation for this discrepancy^41^. Patients with AQP4-IgG-positive NMOSD are considered to have several predisposing medical histories, including malignancies or autoimmune-related systemic diseases such as Sjögren's syndrome^42–45^. Such complications are rare in patients with MS or MOGAD, and this suggests different profiles of immune cells in these diseases. Further studies are required to elucidate the exact roles of neutrophils, monocytes, and lymphocytes in these three neurological diseases.

This study had several limitations. First, there were a few eligible patients with AQP4-IgG-positive NMOSD. The absence of statistically significant differences between patients and matched HCs in total WBC, neutrophil, and monocyte counts could be mainly due to the small number of patients. Further studies with more enrolled patients are required to determine the statistical significance of these cell types. Second, almost all enrolled patients and volunteers in this study were of East Asian ancestry. Therefore, it is uncertain whether the findings of this study can be generalized to other patients from other countries and races. Furthermore, this study did not evaluate the cell types among lymphocytes in detail, such as B cells, CD4^+^ T cells, and CD8^+^ T cells. Further studies evaluating these detailed cellular surface markers to discriminate the cellular types of lymphocytes will be helpful in obtaining deeper insights into the exact role of circulating lymphocytes in the mechanisms of these diseases. Finally, the disease groups and matched control groups in this study did not exclude the individuals with smoking history or comorbidities that may affect the WBC count profiles, such as systemic autoimmune diseases or hematologic diseases. This was because this study aimed to elucidate the overview of the WBC count profiles in the overall patients with AQP4-IgG-positive NMOSD or MOGAD. In future researches, it may be better to exclude the individuals who are with conditions that may affect the WBC count profiles.

In conclusion, the total WBC count, neutrophil count, monocyte count, MLR, and NLR in patients with AQP4-IgG-positive NMOSD and MOGAD were higher than those in their matched controls. Although the total and differential WBC counts did not significantly differ among AQP4-IgG-positive NMOSD, MOGAD, and MS, the relationship between the five differential WBC counts in AQP4-IgG-positive NMOSD may differ from those in the other two diseases. Further studies on the relationship between each differential WBC type and relapsing neurological diseases in the CNS are warranted.

## Supplementary Information

Supplementary Information.

## Data Availability

All data supporting the findings of the present study is shown in Supplementary Table S1.
